# Health Technology Assessment Process for Oncology Drugs: Impact of CADTH Changes on Public Payer Reimbursement Recommendations

**DOI:** 10.3390/curroncol29030127

**Published:** 2022-03-01

**Authors:** Louise Binder, Majd Ghadban, Christina Sit, Kathleen Barnard

**Affiliations:** 1 Save Your Skin Foundation, Penticton, BC V2A 0B2, Canada; kathy@saveyourskin.ca; 2 Canadian Neuroendocrine Tumour Society (CNETS), Cornwall, ON K6J 3R1, Canada; majd.ghadban@protonmail.com; 3 Oncology Patient Advocate, Toronto, ON, Canada; christina.sit@gmail.com

**Keywords:** health technology assessment, HTA oncology treatment access, quality adjusted life years, treatment access, drug access, drug reimbursement, Canadian agency for drugs and technologies in health, CADTH, pan-Canadian oncology drug review, pCODR

## Abstract

Public reimbursement systems face the challenge of balancing provision of needed treatments and the reality of limited resources. Canada has a complex system for drug approval and public reimbursement, with jurisdiction divided between the federal government and the provinces/territories. A pivotal role is that of health technology assessment (HTA), which relies primarily on health economic principles to analyze the value of drugs on a population health basis and make recommendations about public reimbursement. The Canadian Agency for Drugs and Technologies in Health (CADTH) provides recommendations to all provinces but Quebec. This article provides an overview of Canada’s approval and public reimbursement pathway, including the role of HTA and the economic principles on which it relies. Starting in late 2020, CADTH reduced the cost per quality-adjusted life year (QALY) threshold, the metric relied upon in making recommendations to public payers. An analysis of all 56 oncology drug final recommendations issued from January 2020 to January 2022 was conducted and confirms this reduction in the cost per QALY threshold. As a result of this threshold reduction, recommendations to the provinces include, in a number of cases, substantially greater price reductions. The potential implications for successful price negotiation with the pan-Canadian Pharmaceutical Alliance (pCPA), the public negotiating body for the provinces, are discussed.

## 1. Introduction

One of the greatest challenges in Canada’s public drug reimbursement programmes is the ethical tension between potentially competing desired goals. The first is the desire to provide needed treatments and care that save lives and restore reasonable quality of life. The other is the reality that the ability to provide targeted treatments is limited by the fact that funds and other resources to do so are not unlimited.

The implication of this tension includes the reality that individuals may not have access to needed medications through public reimbursement programmes. If they are unable to afford them or do not have private insurance that covers them, they simply will not get them unless they can access them through some type of compassionate access programme.

To make these decisions, Canada has a complex set of systems and processes for the review, recommendation, and approval of treatments for sale and their reimbursement across the country [1]. The complexity in the public sphere is largely, although not entirely, due to the division of jurisdiction between the federal government and the provinces/territories in health, leaving the approval for sale at the federal level and the implementation of treatment reimbursement decisions and the cost of the public reimbursement plans at the provincial/territorial level [2].

In general, the federal government is responsible for funding to ensure access to doctors and hospitals for eligible people living in Canada as a result of the *Canada Health Act* [3]. These funds are generally provided through the Canada Health Transfers [3]. Funding goes to the provinces/territories for implementation. Other than specific groups over which the federal government has jurisdiction, coverage for drugs, biologics, and other treatments and companion diagnostics rests with the provinces/territories [4].

This article provides an overview of the Canadian approval and public reimbursement systems for drugs, including oncology drugs, as well as a description of key health technology assessment (HTA) principles and metrics. The pivotal role of HTA in this system, the pan-Canadian process used to make recommendations to public reimbursers, including provinces, is generally based on health economic principles used to determine value at a population health level. These principles and associated metrics are described.

The Canadian Agency for Drugs and Technologies in Health (CADTH) provides these recommendations to all provinces but Quebec, through the Common Drug Review and the pan-Canadian Oncology Drug Review process for non-oncology and oncology drugs, respectively. In late 2020, CADTH appears to have reduced the cost per quality-adjusted life year (QALY) threshold, the metric relied upon in making recommendations to public payers for oncology drugs. Our analysis of the 56 oncology drugs which received final recommendations from January 2020 to January 2022 confirms this threshold reduction.

This reduction has potential implications for successful price negotiations between public reimbursers and manufacturers conducted by the pan-Canadian Pharmaceutical Alliance (pCPA), the negotiating body for the public payers.

## 2. Materials and Methods

A review of relevant literature was conducted to provide an overview of the pathway from drug development to public access in Canada, with a focus on health technology assessment (HTA), including its underpinning health economic principles, and to provide a brief history of the pan-Canadian Oncology Drug Review (pCODR) process.

A search was conducted on CADTH’s Reimbursement Review Reports database, narrowed to “Oncology Pharmaceuticals” with recommendations issued between January 2020 and January 2022 [5]. The most recent entry included in this search was issued 6 January 2022 at the time of writing, yielding a total of 56 entries. The recommendation reports that included in their titles “Recommendations and Reasons” or “pERC Final Recommendation” were searched for reference to cost per quality-adjusted life year (QALY) threshold values, and the results are tabulated in Table S1 in the Supplementary Materials.

The data in Table S1 include the brand name, generic name, project code, therapeutic area, the date the submission was received, the date the recommendation was issued, the final recommendation, reimbursement conditions where applicable, the referenced cost per QALY threshold, and supporting quotes extracted from the recommendation reports. Where a recommendation report refers to one or more threshold values, a supporting quote was selected to include the referenced threshold values.

Case examples included in the Discussion section were extracted from the Table S1.

The Results section of this article includes a quote from the President and CEO of CADTH from Day 1 of the 20th Annual Market Access Virtual Summit, held on 5 October 2021 [6], organized by the Strategy Institute [7]. To ensure the accuracy of this quote, it was transcribed directly from the session recording on the 20th Annual Market Access Virtual Summit platform. In transcribing the quote, only filler words were removed, retaining the substance of the quote.

To develop the recommendations included in the Conclusion section of this article, relevant literature, including international literature, was reviewed.

## 3. Results

### 3.1. From Drug Development to Public Access—An Overview

**Figure 1:**
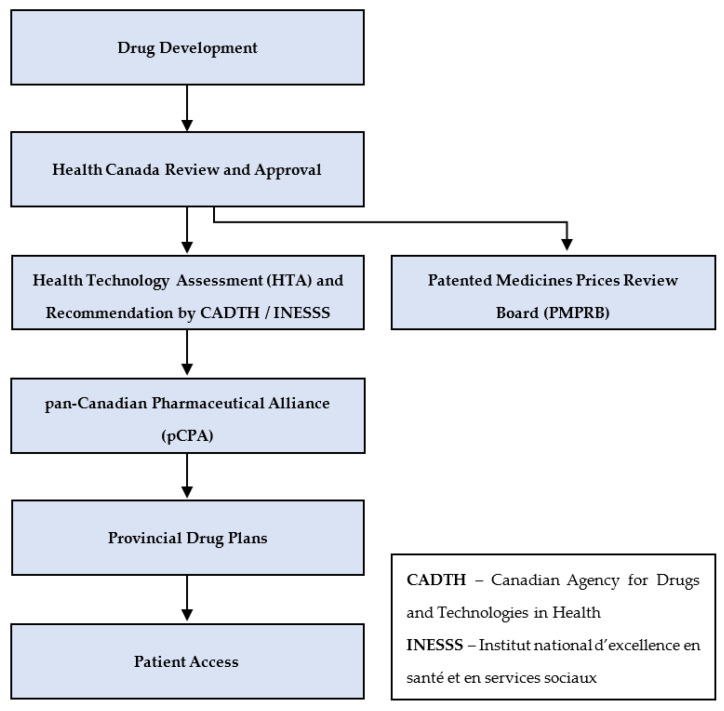
Overview of key steps in the public approval and reimbursement pathway, excluding hospitals. Adapted from [1]. After a drug is developed, the manufacturer submits it to Health Canada for approval for sale [8]. If approved, the manufacturer applies to the Patented Medicines Prices Review Board (PMPRB) for a review of its proposed list price [13]. The manufacturer also generally applies to one or both health technology assessment (HTA) processes in Canada, CADTH for all provinces/territories, except Quebec, which relies on INESSS [1]. If recommended for reimbursement, the manufacturer applies to the pCPA to negotiate a price with the participating provinces/territories. Following successful price negotiations, each province determines if and when to add the product to its public reimbursement plan [7].

Both federal and provincial/territorial decision makers have created departments, agencies, and other bodies to assist in making decisions in health, including decisions regarding public sale and reimbursement of treatments. Figure 1 provides an overview of key steps along that pathway, occurring at the federal, pan-Canadian, and provincial/territorial levels. Details of each level are described below.

#### 3.1.1. Federal Government Processes

After a drug is developed, the manufacturer submits it to Health Canada for approval for sale [8]. Health Canada is a federal body that has within its mandate the role of ensuring treatments approved for sale are safe, effective, and of good quality, with expedited review processes for serious and life-threatening conditions [9,10]. It is moving to a process including Agile Regulations for innovative drugs for serious diseases [11]. It has joined the U.S. Food and Drug Administration (FDA) and certain other international regulators to collaborate in joint drug reviews for cancer, rare diseases, and conditions with limited treatment options, called Project Orbis, allowing for simultaneous review and earlier approval of these medications for sale [12].

If approved, the manufacturer applies to the Patented Medicines Prices Review Board (PMPRB) for a review of its proposed list price. The Patented Medicine Prices Review Board (PMPRB) is a federal agency that determines whether the price a manufacturer proposes as a list price for Canada is “excessive” based on regulations set out in the *Patent Act Regulations* and PMPRB Guidelines [13]. The federal government has announced a third federal government body called the Canada Drug Agency with details of its role to be determined [14].

#### 3.1.2. Pan-Canadian Processes

After approval for sale by Health Canada, the manufacturer also generally applies to health technology assessment (HTA) processes in Canada. The Canadian Agency for Drugs and Technologies in Health (CADTH) makes recommendations to all provinces/territories, except Quebec, about whether public reimbursement plans should reimburse drugs based on determining the “value” on a population health basis [15,16]. Quebec does its review through an agency called Institut national d’excellence en santé et en services sociaux (INESSS) [1]. After receiving a positive recommendation from CADTH, generally conditional on a price reduction and potentially other conditions, most drugs, including oncology drugs, go to the pan-Canadian Pharmaceutical Alliance (pCPA) for price negotiations. The pCPA was created in 2010 by the Council of the Federation to negotiate public drug prices collectively [7]. Provinces may or may not decide to join negotiations. Usually, one province takes the lead on the negotiations. Details of the price agreement are confidential [17,18].

#### 3.1.3. Provinces and Territories

Even if they do join the pCPA negotiations, which they are under no requirement to do, provinces/territories are under no commitment to add the treatment onto their public reimbursement plan immediately, or indeed at any time. Each province makes its own decision about drug reimbursement coverage and any conditions related to this coverage [18].

If the drug is listed on the public formulary, it is reimbursed for patients as determined by the payer. Figure 1 provides an overview of the key steps described above.

### 3.2. Fundamentals of Health Technology Assessment in Public Drug Access

One of the most important aspects in this Byzantine system is the pivotal role of health technology assessment (HTA) bodies in providing drug reimbursement recommendations to public payers based on the value of the product. The Canadian Agency for Drugs and Technologies in Health (CADTH) has the lion’s share of that role in Canada, advising all provinces and territories but Quebec [1]. HTA relies on evidence-based processes to inform decision making in the selection and utilization of health technologies, bridging research and policy. HTA includes health economic models for the most part, and a central economic metric relied upon is the quality-adjusted life year, or QALY [19].

#### 3.2.1. The Science of QALYs: Is a QALY Just a Number?

The QALY was created as a measure of health effectiveness of medical interventions, to be used in cost-effectiveness analyses when allocating limited healthcare resources [20,21,22].

The QALY uses an interval scale to value utility in which death is valued at 0 and a state of perfect health is valued at 1 [23]. The utility is then multiplied by the number of life years gained from a given medical intervention to obtain the QALY (1), or more practically, the number of years lived in perfect health [24].

QALY = years × utility(1)

The utility piece is generally derived from health utilities capturing quality of life, including health-related quality of life (HRQoL weights). Methods for obtaining these utilities include validated direct and indirect approaches [25].

For a hypothetical treatment that extends life by 2 years, lived in less than perfect health ascribed a utility value of 0.5, the QALY would be 1 (2).

2 years × 0.5 = 1 QALY(2)

QALYs play an integrating role, providing an index that combines both quantity and quality of life [24].

#### 3.2.2. Are QALYs the Same across Disease Areas?

An implicit assumption to using QALYs is that all QALYs are of equal societal value, notwithstanding to whom they apply. This, however, fails to recognize different health conditions and personal characteristics, including severity of disease and access to social determinants of health [25]. Although there are certainly some unique challenges to QALYs [26], and they have been criticized on conceptual, operational, and ethical grounds [27,28], none of these is the subject of this review. For public reimbursement planning and administration, QALYs can offer a reliable process for reviews of patented drugs. What is clear is that when using QALYs, a QALY in oncology is not necessarily the same as a QALY in a non-oncology setting [29].

#### 3.2.3. How Much to Pay for a QALY?

Different approaches to health economic evaluation include cost minimization, cost effectiveness, cost utility, cost benefit, and cost consequence analyses [30]. Cost utility analyses play a central role in economic evaluations conducted by CADTH [31]. Using QALYs as a measure of health serves as the basis for the calculating a cost-effectiveness, or cost-utility ratio, in a cost utility analysis. This is useful in comparing the efficiency of different health interventions [32].

When assessing the health economic value, taking into account both quantity and quality, an incremental cost-effectiveness ratio (ICER) is generally calculated [33]. An ICER is the ratio of extra cost per extra unit of health output obtained, and it can be described by the following Equation (3):

(3) $$\mathrm{ICER}=\frac{\mathrm{incremental}\;\cos t}{\mathrm{inremental}\;\mathrm{effectiveness}}=\frac{\cos t\;\mathrm{of}\;\mathrm{new}\;\mathrm{intervention}-\cos t\;\mathrm{of}\;\mathrm{comparator}}{\mathrm{effectiveness}\;\mathrm{of}\;\mathrm{new}\;\mathrm{intervention}-\mathrm{effectiveness}\;\mathrm{of}\;\mathrm{comparator}}$$

QALYs can be used as the desired health output in the calculation of an ICER and can therefore be expressed as cost per incremental gain in QALY (CAD per QALY) [33,34]. Once a cost per QALY has been calculated, the HTA body may compare it against pre-established thresholds to determine the maximum amount of funding per QALY to recommend that a province/territory pays.

There are generally three methods for establishing a cost per QALY threshold. The first and most common is the value displaced, also called opportunity cost, from introducing a new drug. This is based on empirical efforts to estimate the cost effectiveness of services already in the health system that would be displaced at the margin if new services are introduced [29,35]. A second method is the determination of societal willingness to pay for a QALY, and the third is the creation of an objective benchmark for the level of spending relative to gross domestic product (GDP) per capita [29].

### 3.3. History of Oncology HTA in Canada

The role of CADTH in HTA reviews of oncology drugs has changed over time. When CADTH was originally created, oncology drugs were under its purview in the Common Drug Review (CDR) process. A decision to set up a separate oncology HTA process outside CADTH was made between 2006 and 2007 by the Premiers and Ministers and Deputy Ministers of Health for all provinces but Quebec, with distinct processes and a more inclusive approach to health technology assessment for cancer treatments, which became the deliberative framework for reviews. These decisions were made in recognition that the existing HTA review processes were inadequate in delivering consistent, timely, and high-quality reviews for the complexity in the oncology research area [36,37].

Patient groups, individually and in coalition, had been urging the governments to include patient group and individual patient input into this process since the inception of HTA, with the Joint Oncology Drug Review (JODR) and the interim Joint Oncology Drug Reivew (iJODR) the predecessors to the pan-Canadian Oncology Drug Review (pCODR) [37].

In April 2007, the House of Commons Standing Committee on Health heard from witnesses on issues related to the Common Drug Review’s approach to the review of cancer treatments in oncology [38]. Four key issues described from their experiences with CDR in the oncology area were:

1. The need for a distinct process separate from CADTH to address oncology-specific needs;
2. The need for highly credible clinical oncology expertise deeply and transparently integrated within all parts of the process;
3. The need for patient inclusion in the process;
4. The need for recommendations that focus on patient as well as payer needs, with a clear and transparent rationale for those recommendations [38].

The Standing Committee issued its report in December 2007. This report included recognition of the rationale for a review process that was separate from CADTH for oncology treatments. In late 2013, the federal/provincial/territorial Deputy Ministers of Health made the decision to transfer oncology HTA processes back to CADTH effective April 2014 [36]. The rationale for the decision was “to further consolidate policy direction across different drug programs, and improve the pCODR governance structure to ensure its long term viability and sustainability” [39]. The decision was taken to continue to review oncology drugs through the pCODR process that had been in place prior to the transfer. This process involves a deliberative framework, including an analysis of four criteria—i.e., overall clinical benefit, alignment with patient values, cost effectiveness, and feasibility of adoption into the health system. No specific weighting is ascribed to each of these considerations [40]. Patient groups lauded this decision by CADTH.

In September 2020, CADTH announced the alignment between pCODR and CDR. The three CADTH review pathways that existed previously—the Common Drug Review (CDR), the pan-Canadian Oncology Drug Review (pCODR) and the Interim Plasma Product Review—became one procedure [41].

### 3.4. Analysis of CADTH’s Evaluative Process

#### Reduction in Cost per QALY Threshold

Late in 2020, it appears that CADTH generally began using 50,000 CAD per QALY as the assumed threshold in recommendations issued for oncology treatments. There is some evidence to suggest that 50,000 CAD per QALY is the threshold used by the Common Drug Review (CDR), though not explicitly stated [42]. The Common Drug Review (CDR) reviews non-oncology applications.

Previously, the process for reviewing oncology drugs at CADTH, the pan-Canadian Oncology Drug Review process (pCODR), appeared to have used higher cost per QALY thresholds, with some evidence suggesting thresholds of up to 140,000 CAD [43]. There is support for this upper bound of 140,000 CAD incremental cost-effectiveness ratio threshold, as it was relied on in an analysis conducted by a team that included members of the pCODR Expert Review Committee (pERC) [44].

While consultations were held regarding the administrative alignment of the three processes, no announcement or public consultation with stakeholders was undertaken prior to the reduction in the cost per QALY threshold [45]. The President and Chief Executive Officer indicated the reason for this change at a presentation made at the 20th Annual Market Access Virtual Summit on 5 October 2021 [6] as follows:

*“CADTH does its works both independently but in response to what’s being asked of us. Canada has not set a cost effectiveness threshold and so there isn’t common agreement across Canada on what those should be so the 50 QALY, the $50,000 QALY has been selected for consistency across all CADTH products and conditions and it allows our decision makers in the absence of any gradiated (sic) or alternate numbers to be able to compare across different interventions and drugs. However it’s important to also note that we do look at the impact of other potential price reductions as part of our work and those are clearly reported in the documents so although that’s the one that gets the most visibility, all of the other price thresholds are also reflected on in our report. And I think for us the high price reductions if they are sitting in the 80 to 90 percent for the most part are a signal to the pCPA and to jurisdictions that these are products that may have greater complexity, higher uncertainty and may, may result and signal more work to be able to negotiate. As we said, always evolving this place and continuing to work but that sort of is the purpose right now is in the absence of Canada agreeing on any other thresholds, we‘re using the one as sort of a consistent place to be able for people to make judgment but certainly there is more in the reports and so for people to take a look at that as well”.*

**Figure 2:**
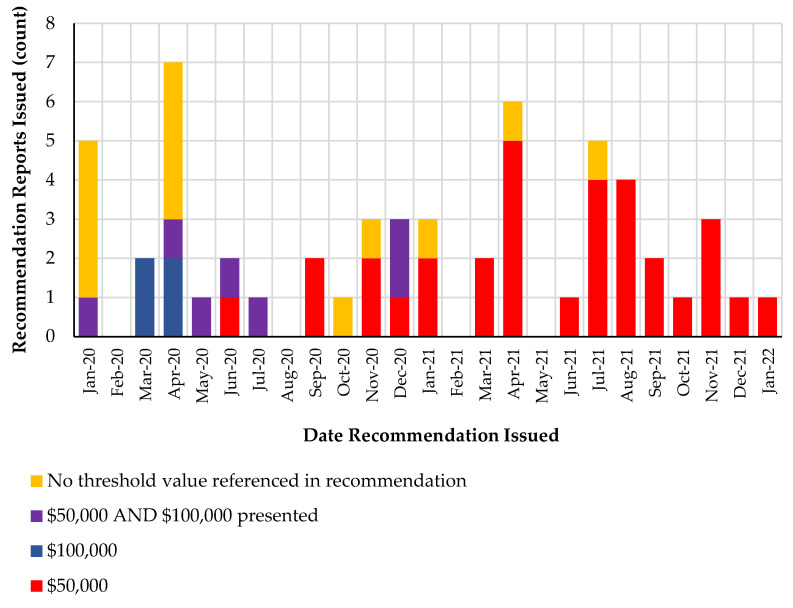
Cost per QALY threshold values referenced in CADTH’s Recommendation Reports for Oncology Pharmaceuticals from January 2020 to January 2022. Source data can be found in Table S1.

After analyzing CADTH’s pCODR recommendation reports from January 2020 to January 2022, it appears that a single threshold of 50,000 CAD cost per QALY was predominantly referred to in its recommendation reports for oncology drugs issued in late 2020 and onward (Figure 2). Additionally, it appears in Figure 2 that none of the recommendation reports issued after December 2020 have reference to multiple thresholds and the price reduction required to meet each threshold value.

## 4. Discussion

### 4.1. Potential Implications of the Threshold Reduction

This begs the question about what has changed in provincial decision making on this issue, since prior to this change the provinces were relying on recommendations based on seemingly higher thresholds for oncology drugs reviewed at pCODR without any public expression of dissatisfaction.

CADTH is an independent, not-for-profit organization responsible for providing Canada’s health care decision-makers with objective evidence to help make informed decisions about the optimal use of health technologies, including drugs and medical devices, in the Canadian healthcare system [15]. Thus, the statement by the President and CEO that it both works independently and “in response to what is being asked of us” is ambiguous at best and does not appear to be aligned with its stated mandate.

As an expert independent health technology assessment body, included in CADTH’s role is advising public payers about the appropriate cost per QALY thresholds to be used in decision making. If the provinces decide not to accept the recommendations for reimbursement following from this analysis, they can deal with it at the pCPA table. Ironically, when CADTH was first created, many patient groups argued that this was just a duplicative process since provinces continued to keep their own drug review bodies. At the time, the response was that over time the provinces would disband these bodies, but in fact, this has generally not occurred in the largest provinces [1].

While CADTH is certainly charged with reconsidering its decisions and changing its recommendations about appropriate thresholds, this should be carried out through evidence-based analyses, including a review of international comparators. Cost per QALY thresholds are not just arbitrary numbers, and as discussed previously, they require an evidence-based approach in their selection. The evidence upon which it has made these changes should have been provided transparently to all stakeholders and introduced only after consultations were conducted.

The implications of this change for oncology HTA recommendations for price reductions may be significant in some cases. While drugs may well still be given approval conditional on price reductions, the required price reductions may be so significant that companies may not submit them to pCPA for negotiation or may not reach a negotiated price that the company considers profitable enough to accept from the public payers. This is not to suggest that the pCPA should not fulfill its mandate of negotiating affordable drugs prices for public plans. Tactics to do so may include agreements including pay-for-performance, risk-sharing, and other strategies for making drugs accessible to patients.

An analysis by Innovative Medicines Canada found that from 2011 to 2018 Canada launched approximately the same number of new drugs as other OECD countries, but its reimbursement rate was appreciably lower, reimbursing 32–45% fewer new medicines. Furthermore, Canada reimburses fewer new drugs than its global counter parts, regardless of special review status, including an oncology designation [46].

Here are some examples of oncology drugs that require profound price reductions using the 50,000 CAD threshold:

Enzalutamide, for metastatic hormone-sensitive prostate cancer (mHSPC), was given a recommendation to reimburse with clinical conditions and/or criteria on 23 September 2020. The reimbursement conditions included cost-effectiveness being improved to an acceptable level, citing a required 75% price reduction to meet a willingness-to-pay threshold of 50,000 CAD per QALY:

*“The CADTH reanalysis results indicated that enzalutamide plus ADT was not cost-effective at a willingness-to-pay threshold of $50,000 per quality-adjusted life-year (QALY), with an incremental cost-effectiveness ratio of $294,805 per QALY at the current price. Based on current list prices, at a willingness-to-pay threshold of $50,000 per QALY, a price reduction of approximately 75% is required”.*

[47]

Venetoclax for acute myeloid leukemia (AML) was given a recommendation to reimburse with clinical conditions and/or criteria on 20 August 2021. The reimbursement conditions indicated that even a 100% reduction in price would not be sufficient to achieve an ICER of 50,000 CAD per QALY:

*“The ICER for venetoclax plus azacitidine is $125,580 per QALY gained when compared to LDAC. A 100% reduction in the price of venetoclax would still not achieve an ICER of $50,000 per QALY compared to LDAC. Azacitidine is more costly than LDAC and would also need to be reduced in price to reach this threshold”.*

[48]

Larotrectinib, for solid tumours with NTRK gene fusion, was given a recommendation to reimburse with clinical conditions and/or criteria on 13 September 2021. The reimbursement conditions included a reduction in price of greater than 90% to be considered cost-effective at the 50,000 CAD threshold:

*“If testing is required to determine eligibility based on NTRK status, then there is no price at which larotrectinib could be considered cost-effective at a $50,000 per QALY threshold. If the cost of testing to determine eligibility based on NTRK status is excluded from the total treatment cost, then larotrectinib would require a price reduction of greater than 90% to be considered cost-effective at a $50,000 per QALY threshold”.*

[49]

Most recently, enfortumab vedotin for locally advanced or metastatic urothelial carcinoma was given a recommendation to reimburse with clinical conditions and/or criteria on 6 January 2022. Reimbursement conditions include a reduction in price, citing that a 93% reduction in price is required to achieve an ICER of 50,000 CAD per QALY:

*“The ICER for enfortumab vedotin is $506,439 when compared with taxanes. A price reduction of 93% would be required for enfortumab vedotin to be able to achieve an ICER of $50,000 per QALY compared to a taxane”.*

[50]

Here is an example where both thresholds of 50,000 CAD and 100,000 CAD were presented in the recommendation reports:

Cemiplimab for advanced cutaneous squamous cell carcinoma was given a recommendation to reimburse with clinical conditions and/or criteria on 22 January 2020. The reimbursement conditions included cost-effectiveness being improved to an acceptable level, citing 40% and 80% reduction requirements to bring the incremental cost-utility ratio (ICUR) to 100,000 CAD and 50,000 CAD per QALY, respectively.

*“In the EGP’s best-case estimate, the incremental cost of cemiplimab was $176,966 and the incremental benefit gain was 1.48 LYs and 1.06 QALYs over a 30-year life-time horizon, for an estimated ICUR of $166,221 per QALY. An upper bound of $223,828 per QALY was achieved with cemiplimab being administered until treatment progression (no capping at 22 or 24 months). The cost of cemiplimab was the main cost driver; and most of the QALY gained (70%) was accrued in the post-progression period and in the extrapolated phase of the model. The deterministic sequential analysis showed that for a willingness-to-pay below $52,539 per QALY, BSC would be the preferred treatment option. For a willingness-to-pay between $52,539 and $161,278 per QALY, chemotherapy would be the preferred option, and that cemiplimab would be the preferred option for a willingness-to-pay above $161,278 per QALY. The price reduction scenarios showed that a 40% price reduction would be needed to bring the ICUR around $100,000 per QALY while an 80% price reduction would be required to bring the ICUR around $50,000 per QALY”.*

[51]

### 4.2. Cost per QALY Thresholds for Decision Making in Comparator Countries

The National Institute for Health and Care Excellence (NICE) in the U.K. has used a threshold of GBP 20,000 to GBP 30,000 per QALY; however, NICE’s threshold can vary depending on the technology. While cost per QALY thresholds are used to guide decision-making in the U.K., they are applied in a flexible manner. This is key when considering the uncertainties involved [29]. An example of this is the end-of-life guidance, which is defined as a treatment given to patients with a prognosis of less than 2 years to live, where the treatment can extend life for 3 or more months. The cost per QALY threshold increases by 2.5-fold, to about GBP 50,000 per QALY. Another example is the using modifiers when opting to approve a drug that exceeds the standard threshold, as put into practice by NICE and the Scottish Medicines Consortium (SMC). These modifiers include improvement in QALY, increased life expectancy, and the absence of other therapeutic options [29].

The Institute for Clinical and Economic Review in the U.S. uses an open and transparent engagement process with stakeholders in the development of its economic models. The Institute for Clinical and Economic Review provides incremental results for USD 50,000, USD 100,000, USD 150,000, and USD 200,000 per QALY and per equal value of life years gained (evLYG), and the institute continues to use USD 100,000 and USD 150,000 per QALY as the standard for health-benefit price benchmarks. The Institute for Clinical and Economic Review is committed to open and transparent engagement with stakeholders in developing their economic models [52].

In the Netherlands, Zorginstituut Nederland, the national healthcare institute, introduced explicit variable willingness to pay values depending on disease severity. The aim was to reflect the judgement of society’s willingness to pay more for patients with conditions of greater need. Three distinct ranges of disease severity were developed and assigned willingness to pay reference values ranging from of EUR 20,000 to EUR 80,000 per QALY gained, based on the severity category in which the disease falls [53].

In Sweden, the Dental and Pharmaceutical Benefits Agency, known as the Tandvårds-och läkemedelsförmånsverket, or TLV for short, assigns a higher priority to diseases of higher severity, and is flexible in setting an accepted cost per QALY depending on the severity of disease. This stems from the TLV’s principles of human dignity/value, need and solidarity, and cost-effectiveness [54,55].

## 5. Conclusions—Recommendations for CADTH

CADTH appears to have made a decision to use a cost per QALY threshold of 50,000 CAD for oncology as a result of the fact that there is no pan-Canadian agreement as to the appropriate cost per QALY threshold. The determination of a QALY and a cost per QALY threshold are not just arbitrary but are evidence based. The concept of HTA and cost per QALY thresholds as economic tools, among others, are certainly reasonable for aiding public reimbursement decision making, but they cannot be taken as a rule. CADTH should take the lead of Health Canada and develop a “Project Orbis” type process with other countries. It should certainly follow the U.S. practice of transparent and open engagement of stakeholders, including representatives of diverse patient populations, in the development of economic models. CADTH’s role is to make recommendations to public payers for reimbursement. Decisions about price are the mandate of the pCPA, which can certainly develop pay-for-performance and risk-sharing agreements that will ensure prices that public reimbursers can accept as creating a sustainable public reimbursement system.

## Data Availability

Not applicable.

## Supplementary Materials

The following supporting information can be downloaded at: https://www.mdpi.com/article/10.3390/curroncol29030127/s1, Table S1: Analysis of CADTH’s Recommendation Reports for Oncology Pharmaceuticals Issued between January 2020 and January 2022. References [47,48,49,50,51] are cited in the supplementary materials.
