# Supplementary material for: Health Technology Assessment Process for Oncology Drugs: Impact of CADTH Changes on Public Payer Reimbursement Recommendations
Source: Curr Oncol. 2022 Mar 1;29(3):1514–26. doi: 10.3390/curroncol29030127 (PMC8947453; doi:10.3390/curroncol29030127)
Supplement: Supplementary file 1 [file curroncol-29-00127-s001.zip › curroncol-1595326-supplementary.pdf]

Table S1. Analysis of CADTH's Recommendation Reports for Oncology Pharmaceuticals Issued between January 2020 and January 2022.

Brand Name	Generic Name	Project Code	Therapeutic Area	Date Submission Received	Date Recommendation Issued	Final Recommendation	Reimbursement Conditions	Referenced cost per QALY Threshold in Recommendation	Supporting Quote for Reference to Threshold	Source
Padcev	enfortumab vedotin	PC0251-000	Locally advanced or metastatic urothelial carcinoma	23-Jun-21	06-Jan-22	Reimburse with clinical criteria and/or conditions	Reimbursement conditions include a reduction in price	$50,000	"The ICER for enfortumab vedotin is $506,439 when compared with taxanes. A price reduction of 93% would be required for enfortumab vedotin to be able to achieve an ICER of $50,000 per QALY compared to a taxane."	[1]
Keytruda	pembrolizumab	PC0250-000	Esophageal carcinoma, gastroesophageal junction adenocarcinoma	26-May-21	20-Dec-21	Reimburse with clinical criteria and/or conditions	Reimbursement conditions include a reduction in price	$50,000	"The ICER for pembrolizumab in combination with 5-FU and cisplatin is $170,819 per QALY when compared with 5-FU plus cisplatin alone. A price reduction of 75% would be required for pembrolizumab to be able to achieve an ICER of $50,000 per QALY compared with 5-FU plus cisplatin."	[2]
Ledaga	chlormethine hydrochloride	PC0242-000	T-cell lymphoma	21-Dec-20	24-Nov-21	Do not reimburse	Not applicable	$50,000	"When CADTH performed exploratory reanalyses assuming confidence in the naive comparison of chlormethine gel and phototherapy, chlormethine gel had a 0.2% probability of being cost-effective at a willingness-to-pay (WTP) threshold of$50,000 per quality-adjusted life-year (QALY) in the population of patients enrolled in Study 201."	[3]
Tukysa	tucatinib	PC0243-000	Advanced or Metastatic Breast Cancer	26-Mar-21	17-Nov-21	Reimburse with clinical criteria and/or conditions	Reimbursement conditions include a reduction in price	$50,000	"The ICER for tucatinib combination therapy is $512,403 per QALY compared to T-DM1 in the second-line setting and $381,429 per QALY compared to trastuzumab with capecitabine in the third-line setting. A price reduction of 48% would be required for tucatinib combination therapy to be able to achieve an ICER of $50,000 per QALY compared to T-DM1 in the second-line setting. A price reduction of 94% would be required for tucatinib combination therapy to be able to achieve an ICER of $50,000 per QALY compared to trastuzumab with capecitabine in the third-line setting."	[4]
Keytruda	pembrolizumab	PC0236-000	Classical Hodgkin lymphoma	29-Jan-21	01-Nov-21	Reimburse with clinical criteria and/or conditions	Reimbursement conditions include a reduction in price	$50,000	"The ICER for pembrolizumab is $733,624 to $2,071,825 per QALY in the adult ASCT-eligible population. Pembrolizumab was less costly and more effective (dominant) when compared with BV in a pediatric ASCT-eligible population. CADTH undertook a price reduction analysis in an adult ASCT-eligible population. This analysis indicated that a 13% to 29% reduction in price is required to achieve an ICER of $50,000 per QALY. The range reflects uncertainty regarding subsequent therapy use. The cost-effectiveness of pembrolizumab is unknown in an ASCT-ineligible population meaning further price reductions may be required."	[5]
Onureg	azacitidine	PC0245-000	Acute myeloid leukemia	01-Mar-21	20-Oct-21	Reimburse with clinical criteria and/or conditions	Reimbursement conditions include a reduction in price	$50,000	"The ICER for oral azacitidine is $355,456 when compared with BSC. A price reduction of at least 85% would be required for oral azacitidine to be able to achieve an ICER of $50,000 per QALY compared to BSC."	[6]
Inqovi	Decitabine-Cedazuridine	PC0228-000	Myelodysplastic Syndromes (MDS)	09-Oct-20	22-Sep-21	Reimburse with clinical criteria and/or conditions	Reimbursement conditions include the following: Decitabine and cedazuridine should be negotiated to provide cost savings to the CADTH-participating-drug programs for adult patients with de novo or secondary MDS who are not considered candidates for hematopoietic stem cell transplantation relative to azacitidine in jurisdictions that fund azacitidine for this indication.	$50,000	"There is substantial clinical and methodological uncertainty surrounding the comparative efficacy of decitabine and cedazuridine with azacitidine, treatment wastage, and administration costs."	[7]
Vitrakvi	larotrectinib	PC0221-000	Solid tumours with NTRK gene fusion	16-Nov-20	13-Sep-21	Reimburse with clinical criteria and/or conditions	Reimbursement conditions include a reduction in price	$50,000	"If testing is required to determine eligibility based on NTRK status, then there is no price at which larotrectinib could be considered cost-effective at a $50,000 per QALY threshold. If the cost of testing to determine eligibility based on NTRK status is excluded from the total treatment cost, then larotrectinib would require a price reduction of greater than 90% to be considered cost-effective at a $50,000 per QALY threshold."	[8]
Vyxeos	daunorubicin and cytarabine	PC0237-000	Acute myeloid leukemia	22-Jan-21	24-Aug-21	Reimburse with clinical criteria and/or conditions	Reimbursement conditions include a reduction in price	$50,000	"The ICER for liposomal daunorubicin and cytarabine is $110,283 per QALY compared with 7 + 3. A price reduction of at least 68% would be required for liposomal daunorubicin and cytarabine to achieve an ICER of $50,000 per QALY compared with 7 + 3, although this is likely underestimated."	[9]
Venclexta	venetoclax	PC0239-000	Acute myeloid leukemia	22-Jan-21	23-Aug-21	Do not reimburse	Not Applicable	$50,000	"Economic evidence suggests that Venclexta plus LDAC is not cost-effective at a willingness-to-pay threshold of $50,000 per quality-adjusted life-year (QALY), even at a 100% reduction in the price of Venclexta."	[10]

Venclexta	venetoclax	PC0238-000	Acute myeloid leukemia	08-Jan-21	20-Aug-21	Reimburse with clinical criteria and/or conditions	Reimbursement conditions include a reduction in price	$50,000	"The ICER for venetoclax plus azacitidine is $125,580 per QALY gained when compared to LDAC. A 100% reduction in the price of venetoclax would still not achieve an ICER of $50,000 per QALY compared to LDAC. Azacitidine is more costly than LDAC and would also need to be reduced in price to reach this threshold."	[11]
Opdivo-Yervoy	nivolumab-ipilimumab	PC0229-000	Malignant Pleural Mesothelioma (MPM)	09-Oct-20	04-Aug-21	Reimburse with clinical criteria and/or conditions	Reimbursement conditions include a reduction in price	$50,000	"Nivolumab in combination with ipilimumab is more costly than pemetrexed in combination with platinum-based chemotherapy. The ICER for nivolumab in combination with ipilimumab was $300,921 per QALY. A price reduction of at least 72% for both nivolumab and ipilimumab is necessary for nivolumab in combination with ipilimumab to be considered cost-effective at a $50,000 per QALY threshold."	[12]
Imfinzi	durvalumab	PC0234-000	Extensive-stage small cell lung cancer	01-Dec-20	27-Jul-21	Reimburse with clinical criteria and/or conditions	Reimbursement conditions include a reduction in price	$50,000	"Durvalumab + EP is more costly than EP alone. The ICER for durvalumab in combination with EP was $441,635 per QALY compared with EP alone. A price reduction of at least 88% for durvalumab is necessary for durvalumab + EP to be considered cost-effective at a $50,000 per QALY threshold."	[13]
Keytruda	pembrolizumab	PC0235-000	Colorectal cancer	30-Nov-20	27-Jul-21	Reimburse with clinical criteria and/or conditions	Reimbursement conditions include a reduction in price	$50,000	"Pembrolizumab is more costly than SOC chemotherapy. A price reduction of at least 21% would be required for pembrolizumab to be considered cost-effective at a WTP threshold of $50,000 per QALY."	[14]
Braftovi and Mektovi	encorafenib and binimetinib	PC0232-000	Advanced Melanoma	16-Dec-20	26-Jul-21	Reimburse with clinical criteria and/or conditions	Reimbursement conditions include the following: Encorafenib in combination with binimetinib should not be more costly than the least costly BRAFi/MEKi combination regimen.	No threshold value referenced in recommendation	Not Applicable	[15]
Braftovi	encorafenib	PC0233-000	Metastatic colorectal cancer	16-Dec-20	26-Jul-21	Reimburse with clinical criteria and/or conditions	Reimbursement conditions include a reduction in price	$50,000	"The ICER for encorafenib in combination with cetuximab is $198,779 when compared with FOLFOX. Given the cost of cetuximab, there is no price for encorafenib at which an ICER of $50,000 could be achieved. If the price of cetuximab was reduced by more than 60%, encorafenib may be able to achieve an ICER of $50,000 per QALY, with a 99% price reduction."	[16]
Unituxin	dinutuximab	PC0222-000	Neuroblastoma	23-Nov-20	23-Jul-21	Reimburse with clinical criteria and/or conditions	Reimbursement conditions include a reduction in price	$50,000	"With price reductions approaching 100%, dinutuximab is not cost-effective at a $50,000 per QALY threshold."	[17]
Inrebic	fedratinib	PC0205-000	Myelofibrosis	05-Nov-20	21-Jun-21	Reimburse with clinical criteria and/or conditions	Reimbursement conditions include the following: The drug plan cost of fedratinib should not exceed the drug plan cost of treatment with the least costly JAK inhibitor reimbursed for the treatment of splenomegaly and/or disease-related symptoms.	$50,000	"At the submitted price, fedratinib ($337.57 per day) is more costly than ruxolitinib ($173.26 per day). Given the lack of direct comparative evidence to compare these 2 treatments, and the uncertainty associated with an indirect comparison of JAK inhibitors used to treat myelofibrosis, there is insufficient evidence to justify a cost premium for fedratinib over the least expensive JAK inhibitor reimbursed for the treatment of splenomegaly and/or disease-related symptoms in adults with intermediate-2 or high-risk primary myelofibrosis, post-polycythemia vera myelofibrosis, or post-essential thrombocythemia myelofibrosis."	[18]
Odomzo	Sonidegib	PC0215-000	Basal Cell Carcinoma	19-Jun-20	29-Apr-21	Do not reimburse	Not Applicable	No threshold value referenced in recommendation	Not Applicable	[19]
Zejula	Niraparib	PC0224-000	First Line OC	21-Sep-20	29-Apr-21	Reimburse with clinical criteria and/or conditions	Reimbursement conditions include a reduction in price	$50,000	"A reduction of 60% in the price of niraparib would be required to be considered cost-effective at a WTP threshold of $50,000 per QALY gained; however, a higher price reduction may be required when considering the treatment mix currently used in clinical practice. Niraparib remains dominated by olaparib (i.e., niraparib was equally effective but more expensive) in the BRCA-mut subgroup in a CADTH scenario reanalysis."	[20]

Alunbrig	Brigatinib	PC0230-000	(ALK)-positive locally advanced (not amenable to curative therapy) or metastatic non-small cell lung cancer (NSCLC)	30-Sep-20	21-Apr-21	Reimburse with clinical criteria and/or conditions	Reimbursement conditions include the following: The public drug plan costs of treatment with brigatinib should not exceed the public drug plan price of alectinib, which is currently reimbursed for ALK inhibitor–naïve locally advanced or metastatic NSCLC.	$50,000	"CADTH reanalyses included estimating DoT for brigatinib by extrapolating time-on-treatment data from the ALTA-1L study data, using non-treatment-specific utility weights provided by the sponsor for each health state, and deriving OS and PFS curves from a published NMA rather than the sponsor's submitted unanchored MAIC. According to the sequential analysis of the CADTH base case, the incremental cost-effectiveness ratio for brigatinib was dominated by alectinib (i.e., more costly, less effective). The probability that brigatinib represented the most cost-effective strategy was 0% at a willingness-to-pay threshold of $50,000 per quality-adjusted life-year. An exploratory analysis conducted using the CADTH base case suggested that a 46% price reduction was necessary for brigatinib to be equivalent in cost to alectinib."	[21]
Lynparza	Olaparib	PC0223-000	metastatic castration-resistant prostate cancer (mCRPC)	22-Sep-20	21-Apr-21	Reimburse with clinical criteria and/or conditions	Reimbursement conditions include a reduction in price	$50,000	"Based on CADTH reanalyses, the incremental cost-effectiveness ratio (ICER)for olaparib versus docetaxel was $459,527 per QALY gained; a 71% price reduction for olaparib is required to achieve an ICER of less than $50,000 per QALY. The CADTH base case is reliant on estimates from the sponsor's indirect treatment comparison regarding the comparative efficacy versus docetaxel and cabazitaxel. As noted by CADTH clinical experts, there is no robust evidence to ascertain which of the agents (i.e., olaparib, docetaxel, cabazitaxel, or radium-223) has superior efficacy. Given the high degree of clinical uncertainty, to ensure cost effectiveness at any willingness-to-pay threshold, a further price reduction may be required so that olaparib costs no more than the lowest cost comparator."	[22]
Polivy	Polatuzumab Vedotin	PC0227-000	Diffuse large B-cell lymphoma (DLBCL)	29-Sep-20	21-Apr-21	Reimburse with clinical criteria and/or conditions	Reimbursement conditions include a reduction in price	$50,000	"CADTH was unable to address several major limitations, including the quality of the comparative data and use of a basket comparator. The issues with the clinical data prohibit a reasonable assessment of cost-effectiveness; as such, a CADTH base case could not be derived. CADTH presented a corrected sponsor's base case, which increased the submitted ICER. In addition, CADTH undertook a series of exploratory reanalyses that suggested that the ICER of pola-BR was likely to be higher than estimated by the sponsor and could range from $67,000 per QALY to $147,000 per QALY. However, this suggests that pola-BR controls the disease better than a basket comparator post-progression, which was considered hypothetical and without biological support by clinical experts consulted by CADTH. Based on this range of exploratory analyses, a price reduction for polatuzumab vedotin of between 35% and 84%would be required for pola-BR to become cost-effective at a willingness-to-pay threshold of $50,000 per QALY compared with the basket comparator. However, the uncertainty identified with the comparative clinical information and modelling approach suggest using caution when interpreting these results."	[23]

Sarclisa	Isatuximab	PC0220-000	Multiple Myeloma	17-Aug-20	01-Apr-21	Reimburse with clinical criteria and/or conditions	Reimbursement conditions include a reduction in price	$50,000	"pERC concluded that, at the submitted price, IsaPd is not cost-effective when compared to Pd. The results of the cost-effectiveness analysis were driven by the high cost of isatuximab and pomalidomide. Even with a price reduction for both isatuximab and pomalidomide, it is highly unlikely that IsaPd would be cost-effective at a willingness-to-pay threshold of $50,000 per quality-adjusted life-year (QALY) gained. The cost-effectiveness of IsaPd compared to other relevant (and lower cost) comparator regimens, such as carfilzomib and dexamethasone (Kd) and Pd plus cyclophosphamide, remains unknown at this time given the lack of evidence on its comparative effectiveness."	[24]
Bavencio	Avelumab	PC0225-000	Urothelial Carcinoma (UC)	18-Sep-20	23-Mar-21	Reimburse with clinical criteria and/or conditions	Reimbursement conditions include the following: Cost-effectiveness is improved to an acceptable level	$50,000	"At a willingness-to-pay threshold of $50,000 per QALY, a price reduction of at least 83% is required for avelumab with BSC to be cost-effective. As there remains some outstanding uncertainty within the model regarding potential cost and health consequences associated with adverse events, subsequent treatment costs and utility post disease progression, the resulting ICER may overestimate the cost-effectiveness of avelumab, and the price reduction may be underestimated."	[25]
Opdivo in combination with Yervoy	Nivolumab in combination with Ipilimumab	PC0218-000	NSCLC	23-Jun-20	04-Mar-21	Reimburse with clinical criteria and/or conditions	Reimbursement conditions include the following: Cost-effectiveness being improved to an acceptable level	$50,000	"To be considered cost-effective at a willingness-to-pay threshold of $50,000 per QALY gained, a price reduction of 28% for both nivolumab and ipilimumab would be required. Given the level of uncertainty associated with the economics findings, pERC considered that a greater price reduction may be required to improve the likelihood that nivolumab/ipilimumab plus PDC is a cost-effective treatment. pERC noted the evidence was applicable to the reimbursement request population and Health Canada-approved population."	[26]
Rozlytrek	Entrectinib	PC0206-000	ROS1-positive NSCLC	08-Jan-20	27-Jan-21	Reimburse with clinical criteria and/or conditions	Reimbursement conditions include the following: Cost-effectiveness being improved to an acceptable level	No threshold value referenced in recommendation	Not Applicable	[27]
Daurismo	Glasdegib	PC0207-000	Acute Myeloid Leukemia (AML)	06-May-20	08-Jan-21	Do not reimburse	Not Applicable	$50,000	"CADTH was unable to determine the cost-effectiveness between these treatments and focused the base case results on the main population with azacitidine only being included as part of exploratory analyses. CADTH reanalyses indicated that glasdegib in combination with LDAC versus LDAC alone was not cost-effective at a willingness-to-pay threshold of $50,000 per quality-adjusted life-year (QALY) gained with an incremental cost-effectiveness ratio of $229,622 per QALY gained at the submitted price. A reduction of 95% in the price of glasdegib would be required for glasdegib in combination with LDAC to be considered cost-effective at a willingness-to-pay threshold of $50,000 per QALY gained."	[28]
Calquence	Acalabrutinib	PC0210-000	Chronic Lymphocytic Leukemia (CLL) / Small Lymphocytic Lymphoma (SLL)	07-Apr-20	08-Jan-21	Reimburse with clinical criteria and/or conditions	Reimbursement conditions include the following: cost-effectiveness improved to an acceptable level	$50,000	"The comparison of ACA with CHL-OBI (and ACA-OBI) using the best available data from the ELEVATE-TN trial suggests that ACA is more effective and more costly than CHL-OBI (incremental cost-effectiveness ratio [ICER] = $65,672 per QALY), and associated with greater QALYs and fewer costs compared with ACA-OBI (i.e., dominant). A price reduction of at least 4% for acalabrutinib is required to achieve an ICER of $50,000 per QALY for ACA compared with CHL-OBI."	[29]

Keytruda	Pembrolizumab	PC0216-000	head and neck squamous cell carcinoma (HNSCC)	01-May-20	22-Dec-20	Reimburse with clinical criteria and/or conditions	Reimbursement conditions include the following: cost-effectiveness being improved to an acceptable level	$50,000 AND $100,000 presented	"At a willingness-to-pay threshold of $50,000 per QALY, a price reduction of at least 49% is required for PEMB-mono to be cost-effective, while a price reduction of at least 67% is required for PEMB-chemo to be cost-effective. At a $100,000 per QALY threshold, a price reduction of at least 19% is required for PEMB-mono to be cost-effective, while a price reduction of at least 37% is required for PEMB-chemo to be cost-effective."	[30]
Adcetris	Brentuximab Vedotin	PC0213-000	Primary cutaneous anaplastic large cell Lymphoma (pcALCL) or CD30-expressing mycosis fungoides (MF)	30-Mar-20	03-Dec-20	Reimburse with clinical criteria and/or conditions	Reimbursement conditions include the following: cost-effectiveness improved to an acceptable level	$50,000 AND $100,000 presented	"The probability that BV was cost-effective at a WTP threshold of $50,000 per QALY gained was 0%. Price reductions of at least 64% and 62% are required for BV to be considered cost-effective at the WTP thresholds of $50,000 per QALY gained and $100,000 per QALY gained, respectively, compared with PC."	[31]
Adcetris	Brentuximab Vedotin	PC0214-000	Hodgkin lymphoma (HL) in combination with doxorubicin, vinblastine, and dacarbazine (AVD)	02-Apr-20	03-Dec-20	Reimburse with clinical criteria and/or conditions	Reimbursement conditions include the following: cost-effectiveness being improved to an acceptable level	$50,000	"A price reduction of at least 53% is required for BV to be considered cost-effective at a WTP threshold of $50,000 per QALY gained. The potential price reduction necessary for BV in combination with AVD to be cost-effective is uncertain, however, given the limitations with the analysis."	[32]
Venclexta	Venetoclax Obinutuzumab	PC0212-000	Obinutuzumab for CLL	17-Apr-20	17-Nov-20	Reimburse with clinical criteria and/or conditions	Reimbursement conditions include the following: cost-effectiveness improves to an acceptable level	No threshold value referenced in recommendation	Not Applicable	[33]
Tecentriq & Avastin	Atezolizumab & Bevacizumab	PC0217-000	Hepatocellular Carcinoma (HCC)	21-May-20	17-Nov-20	Reimburse with clinical criteria and/or conditions	Reimbursement conditions include the following: Cost-effectiveness improves to an acceptable level	$50,000	"pERC deliberated upon the cost-effectiveness of atezolizumab in combination with bevacizumab compared with sorafenib and lenvatinib. In discussing the results of the CADTH base case, pERC noted that the change to the OS extrapolation for atezolizumab plus bevacizumab in the CADTH base case had the greatest impact on model results. pERC felt this change highlighted the uncertainty with the long- term efficacy but also noted that even with optimistic estimates of survival for atezolizumab in combination with bevacizumab, the incremental cost-effectiveness ratio (ICER) was far greater than $50,000 per quality-adjusted life-year (QALY). pERC also highlighted the analysis assessing the impact of using the price of biosimilar bevacizumab, noting atezolizumab plus bevacizumab was still not cost- effective at this lower price. pERC concluded it is highly unlikely that atezolizumab plus bevacizumab would be considered cost-effective at a willingness to pay of $50,000 per QALY even if substantial price reductions were obtained for both atezolizumab and bevacizumab."	[34]
Calquence	Acalabrutinib	PC0211-000	Chronic Lymphocytic Leukemia (CLL)	07-Apr-20	17-Nov-20	Reimburse with clinical criteria and/or conditions	Reimbursement conditions include the following: Cost-effectiveness being improved to an acceptable level.	$50,000	"A price reduction of at least 17% for ACA is required to achieve an ICER of $50,000 per QALY compared with either IDELA-RIT/BEN-RIT, or IDELA-RIT. Compared to VEN-RIT, ACA was dominated (i.e., higher costs and fewer QALYs). A price reduction of more than 80% for ACA is required to achieve an ICER of at $50,000 per QALY compared with VEN-RIT, assuming VEN-RIT is considered a key comparator."	[35]
Blincyto	Blinatumomab	PC0204-000	MRD+ ALL Resubmission	20-Jan-20	29-Oct-20	Reimburse with clinical criteria and/or conditions	Reimbursement conditions include the following: Cost-effectiveness being improved to an acceptable level.	No threshold value referenced in recommendation	Not Applicable	[36]

Xtandi	Enzalutamide	PC0209-000	mHSPC	24-Feb-20	23-Sep-20	Reimburse with clinical criteria and/or conditions	Reimbursement conditions include cost-effectiveness being improved to an acceptable level	$50,000	"The CADTH reanalysis results indicated that enzalutamide plus ADT was not cost-effective at a willingness-to-pay threshold of $50,000 per quality-adjusted life-year (QALY), with an incremental cost-effectiveness ratio of $294,805 per QALY at the current price. Based on current list prices, at a willingness-to-pay threshold of $50,000 per QALY, a price reduction of approximately 75% is required."	[37]
Zejula	Niraparib	PC0203-000	Ovarian Cancer	07-Feb-20	03-Sep-20	Reimburse with clinical criteria and/or conditions	Reimbursement conditions include the following: cost-effectiveness improved to an acceptable level	$50,000	"Price reductions of 76% and 61% would be required for niraparib to be considered cost-effective at a willingness-to-pay threshold of $50,000 per QALY gained in the non-germline BRCA and germline BRCA populations, respectively, when compared with active surveillance."	[38]
Tecentriq & Avastin	Atezolizumab & Bevacizumab	PC0155-000	NSQ-NSCLC	18-Nov-19	03-Jul-20	Do not reimburse	Not Applicable	$50,000 AND $100,000 presented	"The results were primarily driven by the substantially high cost of combined treatment. If the cost of atezolizumab was reduced by 99%, the high cost of even biosimilar bevacizumab prevents the treatment from being cost-effective at even an $100,000 per QALY threshold. Along with a 99% price reduction for atezolizumab, the price of biosimilar bevacizumab would need to be approximately 46% below current list price for the ICER to fall below $100,000 per QALY or approximately 85% to fall below $50,000 per QALY. Overall it is highly unlikely that ABCP would be considered a cost-effective use of Canadian health care resources, at a $50,000 or $100,000 per QALY threshold, even if substantial price reductions were obtained for both atezolizumab and bevacizumab."	[39]
Kisqali	Ribociclib	PC0194-000	HR+, HER2- advanced or metastatic breast cancer	26-Aug-19	04-Jun-20	Reimburse with clinical criteria and/or conditions	Reimbursement conditions include the following: cost-effectiveness improved to an acceptable leve	$50,000 AND $100,000 presented	"In the EGP's best-case estimate, the incremental cost of ribociclib plus NSAI and goserelin was $180,936 and the incremental benefit gain was 1.08 LYs and 0.91 QALYs over a 10-year life-time horizon when compared to NSAI plus goserelin, for an estimated ICUR of $197,832 per QALY. The upper and lower bound of the ICUR estimate were $177,829 per QALY and $386,675 per QALY, respectively. The main factors influencing the extra cost and clinical effect are the time horizon and the extrapolation of PFS data after the end of the trial follow-up. The price reduction scenario analyses showed that a price reduction of 55% or greater would be needed to bring the ICUR lower than $100,000 per QALY and an 85% price reduction would be required to bring the ICUR lower than $50,000 per QALY."	[40]
Adcetris	Brentuximab Vedotin	PC0199-000	peripheral T-cell lymphoma (PTCL)	08-Oct-19	04-Jun-20	Reimburse	Not Applicable	$50,000	"To account for the above limitations, CADTH considered: the inclusion of CHOEP as comparator (assuming the same efficacy as CHOP), alternative long-term extrapolations, inclusion of increased non-cancer mortality, the use of a UK value set applied to EQ-5D data collected during the ECHELON-2 trial, the inclusion of AE-specific disutilities, and a revised time horizon of 42 years (i.e., until the cohort reaches 100 years old). CADTH estimated that the ICER of BV plus CHP compared to CHOP is $79,319 per QALY gained, whereas the ICER of BV plus CHP compared to CHOEP is $72,991 per QALY gained. Price reductions of 30% to 35% would bring the ICER to approximately $50,000 per QALY."	[41]

Xospata	Gilteritinib	PC0202-000		28-Oct-19	20-May-20	Reimburse with clinical criteria and/or conditions	Reimbursement conditions include the following: cost-effectiveness improved to an acceptable level	$50,000 AND $100,000 presented	"To account for these limitations, CADTH considered adding BSC as comparator, alternative salvage chemotherapy treatment distributions based on clinical expert feedback, alternative standardized mortality ratio for long-term survivors based on the literature, exclusion of post-HSCT gilteritinib benefit, and revised dose intensity for oral treatments. Based on probabilistic analysis of CADTH's base-case analysis, BSC had the lowest cost and fewest QALYs followed by salvage chemotherapy and then by gilteritinib. At a willingness-to-pay threshold of less than $98,720 per QALY, BSC is the optimal therapy. Salvage chemotherapy is the optimal therapy if the willingness-to-pay threshold is at least $98,720 but less than $168,451 per QALY gained; and gilteritinib is the optimal therapy at a willingness-to-pay threshold of at least $168,451. When using the CADTH base case, approximately 40% and 90% price reductions of gilteritinib would be required to bring the ICER down to around $100,000 and $50,000 per QALY, respectively."	[42]
Nubeqa	Darolutamide	PC0196-000	non-metastatic castration resistant prostate cancer (nmCRPC)	27-Aug-19	22-Apr-20	Reimburse with clinical criteria and/or conditions	Reimbursement conditions include the following: cost-effectiveness being improved to an acceptable level	No threshold value referenced in recommendation	Not Applicable	[43]
Cabometyx	Cabozantinib	PC0186-000	HCC	16-Oct-19	22-Apr-20	Reimburse with clinical criteria and/or conditions	Reimbursement conditions include the following: Cost-effectiveness being improved to an acceptable level	No threshold value referenced in recommendation	Not Applicable	[44]
Erleada	Apalutamide	PC0200-000	metastatic castration-sensitive prostate cancer (mCSPC)	15-Oct-19	22-Apr-20	Reimburse with clinical criteria and/or conditions	Reimbursement conditions include the following: cost-effectiveness improved to an acceptable level.	$50,000 AND $100,000 presented	"The CADTH reanalysis results aligned with the sponsor's base-case results, indicating that apalutamide plus ADT is extendedly dominated by docetaxel plus ADT and abiraterone acetate with prednisone plus ADT. Price reductions can improve the cost-effectiveness of apalutamide plus ADT in patients with mCSPC, if a decision-maker's willingness to pay is $100,000 and $50,000 per quality-adjusted life-year, approximate price reductions between 60% to 70% and 80%, respectively, are required. Several limitations were identified that could not be addressed by CADTH; most notably, the model structure precluded CADTH from exploring the downstream impact of subsequent treatment and the impact of treatment effect waning."	[45]
Kisqali	Ribociclib with Fulvestrant	PC0195-000	+Fulvestrant for HR+, HER2-advanced or metastatic breast cancer	26-Aug-19	22-Apr-20	Reimburse with clinical criteria and/or conditions	Reimbursement conditions include the following: cost-effectiveness improved to an acceptable level	$100,000	"In the EGP's best-case estimate (full trial population), the incremental cost of ribociclib plus fulvestrant was $137,857 and the incremental benefit gain was 0.98 LYs and 0.80 QALYs over a 10-year life-time horizon when compared to fulvestrant alone, for an estimated ICUR of $171,723 per QALY with a range between $157,226 per QALY and $370,710 per QALY. The main factors influencing the extra cost and clinical effect are time horizon and the extrapolation of PFS data after the end of the trial follow-up. The price reduction scenario analyses showed that a price reduction of 50% or greater would be needed to bring the ICUR lower than $100,000 per QALY."	[46]
Rydapt	Midostaurin	PC0193-000	Systemic Mastocytosis	13-Aug-19	02-Apr-20	Do not reimburse	Not Applicable	No threshold value referenced in recommendation	Not Applicable	[47]
Mylotarg	Gemtuzumab Ozogamicin	PC0190-000	Acute Myeloid Leukemia (AML)	09-Aug-19	02-Apr-20	Reimburse	Not Applicable	No threshold value referenced in recommendation	Not Applicable	[48]

Keytruda	Pembrolizumab	PC0185-000	Renal Cell Carcinoma (RCC)	02-Aug-19	02-Apr-20	Reimburse with clinical criteria and/or conditions	Reimbursement conditions include the following: cost-effectiveness being improved to an acceptable level	$100,000	"pERC noted that the EGP's reanalysis of the incremental cost-utility ratio (ICUR) was higher than the sponsor's submitted ICUR of pembrolizumab plus axitinib versus sunitinib. pERC agreed with the EGP reanalysis of waning the treatment effect from 15 years to five years and anchoring the utilities of health states. pERC noted that to achieve an ICUR of approximately $100 000 per quality-adjusted life-year (QALY) for the entire patient population (all IMDC risk categories) of advanced RCC, a price reduction of 75% of pembrolizumab would be required when compared with sunitinib. Therefore, pERC concluded that at the submitted price, pembrolizumab plus axitinib could not be considered cost effective."	[49]
Lonsurf	Trifluridine-Tipiracil	PC0197-000	Gastric Cancer	03-Sep-19	24-Mar-20	Reimburse with clinical criteria and/or conditions	Reimbursement conditions include the following: Cost-effectiveness being improved to an acceptable level.	$100,000	"pERC considered the uncertainties in the model inputs addressed by the EGP and noted that based on 5,000 iterations, the EGP's probabilistic estimate of the ICER of trifluridine-tipiracil plus BSC is $174,465/quality-adjusted life-year (QALY), which differed from the sponsor's best estimate of $150,529/QALY. The EGP made the following changes to the model to address some of its limitations: setting the dose intensity to 100% to capture the full cost of the dosage, adding the additional institution and dispensing fees costs, selecting a five-year time horizon, and changing the frequency of oncology visits and diagnostic testing to annual visits for those with progressing disease. The EGP conducted price reduction scenarios to assess the impact of a change to the incremental cost-utility ratio based on a change to the price of trifluridine tipiracil. From these analyses, it was concluded that a price reduction of 50% to 75% would be necessary to achieve an ICER value of below $100,000 QALY."	[50]
Darzalex	Daratumumab	PC0189-000	Rd for MM	17-Jul-19	05-Mar-20	Reimburse with clinical criteria and/or conditions	Reimbursement conditions include the following: Cost-effectiveness being improved to an acceptable level	$100,000	"From these analyses, it was concluded that an incremental cost-effective review (ICER) around $100,000 QALY could not be achieved even with a price reduction of 95%. pERC noted that this was most likely a result of the high cost of daratumumab as well as the use of daratumumab regimens in subsequent lines of treatment in the comparator arms. pERC noted the EGP's lower and upper bounds for the best case estimate which were about three times higher than the sponsor's submitted ICER. pERC concluded that at the submitted price DRd could not be considered cost-effective compared with VMP, CyBorD, or Rd. Given that pERC concluded that there is a net clinical benefit of DRd compared with Rd in this setting, jurisdictions may want to consider pricing arrangements and/or cost structures that would improve the cost-effectiveness and affordability of daratumumab compared with other treatment options for multiple myeloma."	[51]
Lorbrena	Lorlatinib	PC0183-000	Non-Small Cell Lung Cancer (NSCLC)	11-Jun-19	30-Jan-20	Do not reimburse	Not Applicable	No threshold value referenced in recommendation	Not Applicable	[52]
Tecentriq	Atezolizumab	PC0156-000	Small Cell Lung Cancer (SCLC)	04-Mar-19	30-Jan-20	Do not reimburse	Not Applicable	No threshold value referenced in recommendation	Not Applicable	[53]
Kadcyla	Trastuzumab Emtansine	PC0182-000	Early Breast Cancer (EBC)	02-Jul-19	22-Jan-20	Reimburse	Not Applicable	No threshold value referenced in recommendation	Not Applicable	[54]

Libtayo	Cemiplimab	PC0187-000	Advanced Cutaneous Squamous Cell Carcinoma (CSCC)	09-Jul-19	22-Jan-20	Reimburse with clinical criteria and/or conditions	Reimbursement conditions include the following: Cost-effectiveness is improved to an acceptable level.	$50,000 AND $100,000 presented	"In the EGP's best-case estimate, the incremental cost of cemiplimab was $176,966 and the incremental benefit gain was 1.48 LYs and 1.06 QALYs over a 30-year life-time horizon, for an estimated ICUR of $166,221 per QALY. An upper bound of $223,828 per QALY was achieved with cemiplimab being administered until treatment progression (no capping at 22 or 24 months). The cost of cemiplimab was the main cost driver; and most of the QALY gained (70%) was accrued in the post-progression period and in the extrapolated phase of the model. The deterministic sequential analysis showed that for a willingness-to pay below $52,539 per QALY, BSC would be the preferred treatment option. For a willingness-to-pay between $52,539 and $161,278 per QALY, chemotherapy would be the preferred option, and that cemiplimab would be the preferred option for a willingness-to-pay above $161,278 per QALY. The price reduction scenarios showed that a 40% price reduction would be needed to bring the ICUR around $100,000 per QALY while an 80% price reduction would be required to bring the ICUR around $50,000 per QALY."	[55]
Keytruda	Pembrolizumab	PC0176-000	Squamous NSCLC	08-Feb-19	03-Jan-20	Reimburse with clinical criteria and/or conditions	Reimbursement conditions include the following: cost-effectiveness being improved to an acceptable level	No threshold value referenced in recommendation	Not Applicable	[56]

References

1. CADTH Reimbursement Recommendation - Enfortumab Vedotin (Padcev) Available online: https://www.cadth.ca/sites/default/files/DRR/2022/PC0251REC-Padcev Final-meta.pdf (accessed on 27 January 2022).

2. CADTH Reimbursement Recommendation - Pembrolizumab (Keytruda) Available online: https://www.cadth.ca/sites/default/files/DRR/2021/PC0250 Keytruda - CADTH Final Rec.pdf (accessed on 27 January 2022).

3. CADTH Reimbursement Recommendation - Chlormethine Gel (Ledaga) Available online: https://www.cadth.ca/sites/default/files/DRR/2021/PC0242 Ledaga - CADTH Final Rec-meta.pdf (accessed on 27 January 2022).

4. CADTH Reimbursement Recommendation - Tucatinib (Tukysa) Available online: https://cadth.ca/sites/default/files/DRR/2021/PC0243 Tukysa - CADTH Final Rec_Final.pdf (accessed on 27 January 2022).

5. CADTH Reimbursement Recommendation - Pembrolizumab (Keytruda) Available online: https://www.cadth.ca/sites/default/files/DRR/2021/PC0236 Keytruda - CADTH Final Rec Final.pdf (accessed on 27 January 2022).

6. CADTH Reimbursement Recommendation - Azacitidine (Onureg) Available online: https://cadth.ca/sites/default/files/DRR/2021/PC0245 Onureg - CADTH Final Rec.pdf (accessed on 27 January 2022).

7. Decitabine-Cedazuridine (Inqovi) for MDS - PERC Final Recommendation Available online: https://www.cadth.ca/sites/default/files/pcodr/Reviews2021/10228Decitabine-CedazuridineMDS_FnRec_REDACT_Post22Sep2021_final.pdf (accessed on 27 January 2022).

8. CADTH Reimbursement Recommendation - Larotrectinib (Vitrakvi) Available online: https://www.cadth.ca/sites/default/files/DRR/2021/PC0221 Vitrakvi - CADTH Final Rec KG_NA_Corrected-meta.pdf (accessed on 27 January 2022).

9. CADTH Reimbursement Recommendation - Daunorubicin and Cytarabine (Vyxeos) Available online: https://cadth.ca/sites/default/files/DRR/2021/PC0237 Vyxeos - CADTH Final Rec.pdf (accessed on 27 January 2022).

10. CADTH Reimbursement Recommendation - Venetoclax (Venclexta) Available online: https://www.cadth.ca/sites/default/files/DRR/2021/PC0239 Venclexta - CADTH Final Rec.pdf (accessed on 27 January 2022).

11. CADTH Reimbursement Recommendation - Venetoclax (Venclexta) Available online: https://cadth.ca/sites/default/files/DRR/2021/PC0238 Venclexta (AZA) - Final Rec.pdf (accessed on 27 January 2022).

12. CADTH Reimbursement Recommendation - Nivolumab In Combination With Ipilimumab (Opdivo-Yervoy) Available online: https://www.cadth.ca/sites/default/files/pcodr/Reviews2021/PC0229 Opdivo-Yervoy Final Rec.pdf (accessed on 27 January 2022).

13. CADTH Reimbursement Recommendation - Durvalumab (Imfinzi) Available online: https://cadth.ca/sites/default/files/DRR/2021/PC0234 Imfinzi - CADTH Final Rec.pdf (accessed on 27 January 2022).

14. CADTH Reimbursement Recommendation -Pembrolizumab (Keytruda) Available online: https://cadth.ca/sites/default/files/attachments/2021-07/PC0235 Keytruda - CADTH Final Rec revised_1.pdf (accessed on 27 January 2022).

15. CADTH Reimbursement Recommendation - Encorafenib (Braftovi) in Combination With Binimetinib (Mektovi) Available online: https://www.cadth.ca/sites/default/files/pcodr/Reviews2021/PC0232 Braftovi and Mektovi Final.pdf (accessed on 27 January 2022).

16. CADTH Reimbursement Recommendation - Encorafenib (Braftovi) Available online: https://cadth.ca/sites/default/files/DRR/2021/PC0233 Braftovi - CADTH Final Rec.pdf (accessed on 27 January 2022).

17. CADTH Reimbursement Recommendation - Dinutuximab (Unituxin) Available online: https://cadth.ca/sites/default/files/pcodr/Reviews2021/PC0222 Unituxin - CADTH Final Rec v4.pdf (accessed on 27 January 2022).

18. CADTH Reimbursement Recommendation - Fedratinib (Inrebic) Available online: https://www.cadth.ca/sites/default/files/attachments/2021-06/CADTH_reimbursement_recommendation_fedratinib_%28inrebic%29_comp.pdf (accessed on 27 January 2022).

19. Sonidegib (Odomzo) BCC – PERC Final Recommendation Available online: https://www.cadth.ca/sites/default/files/pcodr/Reviews2021/10215SonidegibBCC_fnRec_Post29Apr2021_final.pdf (accessed on 27 January 2022).

20. Niraparib (Zejula) First Line OC – PERC Final Recommendation Available online: https://www.cadth.ca/sites/default/files/pcodr/Reviews2021/10224NiraparibOC_fnRec_pERC Chair Approved_Post29Apr2021_final.pdf (accessed on 27 January 2022).

21. Brigatinib (Alunbrig) ALK + NSCLC – PERC Final Recommendation Available online: https://www.cadth.ca/sites/default/files/pcodr/Reviews2021/10230BrigatinibALK%2BNSCLC_fnRec_EC21Apr2021_final.pdf (accessed on 27 January 2022).

22. Olaparib (Lynparza) MCRPC – PERC Final Recommendation Available online: https://www.cadth.ca/sites/default/files/pcodr/Reviews2021/10223OlaparibmCRPC_fnRec_REDACT_EC21Apr2021_final.pdf (accessed on 27 January 2022).

23. Polatuzumab Vedotin (Polivy) DLBC – PERC Final Recommendation Available online: https://www.cadth.ca/sites/default/files/pcodr/Reviews2021/10227PolatuzumabDLBCL_fnRec_EC21Apr2021_final.pdf (accessed on 27 January 2022).

24. Isatuximab (Sarclisa) – PERC Final Recommendation Available online: https://www.cadth.ca/sites/default/files/pcodr/Reviews2021/10220IsatuximabMM_fnRec_pERC Chair Approved_Post01Apr2021_final.pdf (accessed on 27 January 2022).

25. Avelumab (Bavencio) UC – PERC Final Recommendation Available online: https://www.cadth.ca/sites/default/files/pcodr/Reviews2021/10225AvelumabUC_FnRec_REDACT_EC23Mar2021_final.pdf (accessed on 27 January 2022).

26. Nivolumab-Ipilimumab NSCLC – PERC Final Recommendation Available online: https://www.cadth.ca/sites/default/files/pcodr/Reviews2021/10218Nivolumab-IpilimumabNSCLC_FnRec_pERC Chair Approved_redact Post04Mar2021_final.pdf (accessed on 27 January 2022).

27. Entrectinib (Rozlytrek) ROS1-Positive NSCLC – PERC Final Recommendation Available online: https://www.cadth.ca/sites/default/files/pcodr/Reviews2021/10206EntrectinibROS1NSCLC_FnRec_EarlyConv_ApprovedbyChair_EC27Jan2021_final.pdf (accessed on 27 January 2022).

28. Glasdegib (Daurismo) AML – PERC Final Recommendation Available online: https://www.cadth.ca/sites/default/files/pcodr/Reviews2020/10207GlasdegibAML_FnRec_approvedbyChair_REDACT_Post08Jan2021_final.pdf (accessed on 27 January 2022).

29. Acalabrutinib (Calquence) CLL (Previously Untreated) – PERC Final Recommendation Available online: https://www.cadth.ca/sites/default/files/pcodr/Reviews2020/10210AcalabrutinibCLL%28previously untreated%29_FnRec_pERC Chair Approved_REDACT_Post08Jan2021_final.pdf (accessed on 27 January 2022).

30. Pembrolizumab (Keytruda) HNSCC – PERC Final Recommendation Available online: https://www.cadth.ca/sites/default/files/pcodr/Reviews2020/10216PembrolizumabHNSCC_FnRec_EC22Dec2020_final.pdf (accessed on 27 January 2022).

31. Brentuximab Vedotin (Adcetris) PcALCL MF – PERC Final Recommendation Available online: https://www.cadth.ca/sites/default/files/pcodr/Reviews2020/10213BrentuximabVedotinpcALCLMF_fnRec_Post03Dec2020_final.pdf (accessed on 27 January 2022).

32. Brentuximab Vedotin (Adcetris) HL AVD – PERC Final Recommendation Available online: https://www.cadth.ca/sites/default/files/pcodr/Reviews2020/10214BrentuximabVedotinHLAVD_FnRec_approvedbyChair_REDACT_Post03Dec2020_final.pdf (accessed on 27 January 2022).

33. VENETOCLAX (VENCLEXTA) OBINUTUZUMAB CLL – PERC Final Recommendation Available online: https://www.cadth.ca/sites/default/files/pcodr/Reviews2020/10212VenetoclaxObinutuzumabCLL_fnRec_EC_Post17Nov2020_final.pdf (accessed on 27 January 2022).

34. Atezolizumab-Bevacizumab HCC – PERC Final Recommendation Available online: https://www.cadth.ca/sites/default/files/pcodr/Reviews2020/10217AtezolizumabBevacizumabHCC_fnRec_EC_Post17Nov2020_final.pdf (accessed on 27 January 2022).

35. Acalabrutinib (Calquence) CLL – PERC Final Recommendation Available online: https://www.cadth.ca/sites/default/files/pcodr/Reviews2020/10211AcalabrutinibCLL_fnRec_REDACT_EC_Post17Nov2020_final.pdf (accessed on 27 January 2022).

36. Blinatumomab (Blincyto) MRD+ BCP ALL – PERC Final Recommendation Available online: https://www.cadth.ca/sites/default/files/pcodr/Reviews2020/10204BlinatumomabMRD%2BBCPALL_fnREC_approvedbyChair_REDACT_Post29Oct2020_final.pdf (accessed on 27 January 2022).

37. Enzalutamide (Xtandi) MCSPC – PERC Final Recommendation Available online: https://www.cadth.ca/enzalutamide-xtandi-metastatic-castration-sensitive-prostate-cancer-details (accessed on 27 January 2022).

38. Niraparib (Zejula) OC– PERC Final Recommendation Available online: https://www.cadth.ca/sites/default/files/pcodr/Reviews2020/10203NiraparibOC_fnRec_pERC Chair Approved_Post_03Sep2020_final.pdf (accessed on 27 January 2022).

39. Atezolizumab-Bevacizumab NSQ-NSCLC – PERC Final Recommendation Available online: https://www.cadth.ca/sites/default/files/pcodr/Reviews2020/10155AtezolizumabBevacizumabNSQ-NSCLC_FinalRec_Post03July2020_final_Redacted.pdf (accessed on 27 January 2022).

40. Ribociclib (Kisqali) HR+ HER2- MBC – PERC Final Recommendation Available online: https://www.cadth.ca/sites/default/files/pcodr/Reviews2020/10194RibociclibMBC_FnRec_Chair Approved_Post04Jun2020_final.pdf (accessed on 27 January 2022).

41. Brentuximab Vedotin (Adcetris) PTCL– PERC Final Recommendation Available online: https://www.cadth.ca/sites/default/files/pcodr/Reviews2020/10199BrentuximabVedotinPTCL_FnRec_approved_Post04Jun2020_final.pdf (accessed on 27 January 2022).

42. Gilteritinib (Xospata) for Acute Myeloid Leukemia – PERC Final Recommendation Available online: https://www.cadth.ca/sites/default/files/pcodr/Reviews2020/10202GilteritinibAML_FnRec_REDACT_EarlyConv_20May2020_final.pdf (accessed on 27 January 2022).

43. Darolutamide (Nubeqa) NmCRPC – PERC Final Recommendation Available online: https://www.cadth.ca/sites/default/files/pcodr/Reviews2020/10196DarolutamidenmCRPC_fnRec_REDACT_EC_22Apr2020_final.pdf (accessed on 27 January 2022).

44. Cabozantinib (Cabometyx) HCC – PERC Final Recommendation Available online: https://www.cadth.ca/sites/default/files/pcodr/Reviews2020/10186CabozantinibHCC_FnRec_EC_22Apr2020_final.pdf (accessed on 27 January 2022).

45. Apalutamide (Erleada) MCSPC – PERC Final Recommendation Available online: https://www.cadth.ca/sites/default/files/pcodr/Reviews2020/10200ApalutamidemCSPC_fnRec_ChairApproved_EarlyConv_22Apr2020_final.pdf (accessed on 27 January 2022).

46. Ribociclib (Kisqali) Fulvestrant HR+ HER2- MBC – PERC Final Recommendation Available online: https://www.cadth.ca/sites/default/files/pcodr/Reviews2020/10195RibociclibFulvestrantMBC_fnRec_pERC Chair Approved_22April2020_final.pdf (accessed on 27 January 2022).

47. Midostaurin (Rydapt) Systemic Mastocytosis – PERC Final Recommendation Available online: https://www.cadth.ca/sites/default/files/pcodr/Reviews2020/10193MidostaurinSM_fnRec_approvedbyChair_REDACT_Post02Apr2020_draft.revised.pdf (accessed on 27 January 2022).

48. Gemtuzumab Ozogamicin (Mylotarg) AML – PERC Final Recommendation Available online: https://www.cadth.ca/sites/default/files/pcodr/Reviews2020/10190GemtuzumabOzogamacinAML_FnRec_2020-04-01_approvedbyChair_Post02Apr2020_final.pdf (accessed on 27 January 2022).

49. Pembrolizumab (Keytruda) RCC – PERC Final Recommendation Available online: https://www.cadth.ca/sites/default/files/pcodr/Reviews2020/10185PembrolizumabRCC_fnRec_2020-03-31_ApprovedpERCChair_Post02Apr2020_final.pdf (accessed on 27 January 2022).

50. Trifluridine-Tipiracil (Lonsurf) for Gastric Cancer – PERC Final Recommendation Available online: https://www.cadth.ca/sites/default/files/pcodr/Reviews2020/10197TrifluridineTipiracilGC_fnRec_EarlyConv_ApprovedbypERCChair_Post024Mar2020_final.pdf (accessed on 27 January 2022).

51. Daratumumab (Darzalex)+Rd for MM - PERC Final Recommendation Available online: https://www.cadth.ca/sites/default/files/pcodr/Reviews2020/10189DaratumumabNDMM_fnRec_2020-03-03_ApprovedbyChair_Post_05Mar2020_final.pdf (accessed on 27 January 2022).

52. Lorlatinib (Lorbrena) NSCLC – PERC Final Recommendation Available online: https://www.cadth.ca/sites/default/files/pcodr/Reviews2020/10183LorlatinibNSCLC_FnRec_ApprovedbyChair_Post_30Jan2020_final.pdf (accessed on 27 January 2022).

53. Atezolizumab (Tecentriq) SCLC – PERC Final Recommendation Available online: https://www.cadth.ca/sites/default/files/pcodr/Reviews2020/10156AtezolizumabSCLC_FnRec_ProceduralReview_approvedbyChair_Post_30Jan2020_final.pdf (accessed on 27 January 2022).

54. Trastuzumab Emtansine (Kadcyla) EBC – PERC Final Recommendation Available online: https://www.cadth.ca/sites/default/files/pcodr/Reviews2020/10182TrastuzumabEmtansineEBC_fnRec_ChairApproved_EarlyConv_22Jan2020_final.pdf (accessed on 27 January 2022).

55. Cemiplimab (Libtayo) CSCC – PERC Final Recommendation Available online: https://www.cadth.ca/sites/default/files/pcodr/Reviews2020/10187CemiplimabCSCC_fnRec_REDACT_EarlyConv_22Jan2020_final.pdf (accessed on 27 January 2022).

56. Pembrolizumab (Keytruda) Squamous NSCLC – PERC Final Recommendation Available online: https://www.cadth.ca/sites/default/files/pcodr/Reviews2019/10176PembrolizumabSQ-NSCLC_fnRec_NOREDACT_ApprovedbyChair_Post_03Jan2020_final.pdf (accessed on 27 January 2022).
